# The Association of the Immune Response Genes to Human Papillomavirus-Related Cervical Disease in a Brazilian Population

**DOI:** 10.1155/2013/146079

**Published:** 2013-07-08

**Authors:** Amanda Vansan Marangon, Gláucia Andreia Soares Guelsin, Jeane Eliete Laguila Visentainer, Sueli Donizete Borelli, Maria Angélica Ehara Watanabe, Márcia Edilaine Lopes Consolaro, Katiany Rizzieri Caleffi-Ferracioli, Cristiane Conceição Chagas Rudnick, Ana Maria Sell

**Affiliations:** ^1^Campinas State University, Rua Carlos Chagas 480, Barão Geraldo, 13083878 Campinas, SP, Brazil; ^2^Basic Health Sciences Department, Maringa State University, Avenue Colombo 5790, 87020900 Maringá, PR, Brazil; ^3^Pathology Sciences Department, Londrina State University, Rodovia Celso Garcia Cid (PR 445), 86051-970 Londrina, PR, Brazil; ^4^Department of Clinical Analysis and Biomedicine, Maringa State University, Avenue Colombo 5790, 87020900 Maringá, PR, Brazil; ^5^Program of Biosciences Applied to Pharmacy, Department of Clinical Analysis and Biomedicine, Maringa State University, Avenue Colombo 5790, 87020900 Maringá, PR, Brazil

## Abstract

The genetic variability of the host contributes to the risk of human papillomavirus (HPV)-related cervical disease. Immune response genes to HPV must be investigated to define patients with the highest risk of developing malignant disease. The aim of this study was to investigate the association of polymorphic immune response genes, namely *KIR*, HLA class I and II, and single-nucleotide polymorphisms (SNPs) of cytokines with HPV-related cervical disease. We selected 79 non-related, admixed Brazilian women from the state of Paraná, southern region of Brazil, who were infected with high carcinogenic risk HPV and present cervical intraepithelial neoplasia grade 3 (CIN3), and 150 HPV-negative women from the same region matched for ethnicity. *KIR* genes were genotyped using an in-house PCR-SSP. HLA alleles were typed using a reverse sequence-specific oligonucleotide technique. SNPs of *TNF −308G>A, IL6 −174G>C, IFNG +874T>A, TGFB1 +869T>C +915G>C,* and *IL10 −592C>A −819C>T −1082G>A* were evaluated using PCR-SSP. The *KIR* genes were not associated with HPV, although some pairs of i(inhibitory)KIR-ligands occurred more frequently in patients, supporting a role for NK in detrimental chronic inflammatory and carcinogenesis. Some HLA haplotypes were associated with HPV. The associations of *INFG* and *IL10* SNPs potentially reflect impaired or invalid responses in advanced lesions.

## 1. Introduction

Human papillomavirus (HPV) infections occur frequently in healthy individuals, and high carcinogenic risk (HR) HPV types are a major causal factor for cervical cancer. Persistent infection with one among approximately 15 genotypes of carcinogenic HPV causes almost all cases of cervical cancer; type 16 and HPV-18 account for more than 70% of the cervical cancers detected worldwide. Despite being considered a preventable disease, cervical cancer remains the second most common malignancy among women worldwide, with a higher incidence in underdeveloped countries [1, 2].

The major mechanisms by which HPV contribute to neoplastic initiation and progression involve the activity of two viral oncoproteins, E6 and E7, which interfere with the critical cell cycle tumor suppressive proteins p53 and retinoblastoma (Rb). However, HPV infection alone is not sufficient to induce malignant transformation. The multistep process of tumor formation requires the contribution of other significant cofactors, such as individual genetic variations, intratypic HPV variability, and environmental factors [1, 2]. The genetic variability of the host also plays a role in the risk of developing cervical cancer, especially variability of genes that control the immune response. These highly polymorphic genes are important risk determinants of HPV persistence and disease progression. 

The innate immune system comprises the first line of defense following HPV infection. It provides nonspecific protection and enhances the adaptive immune response [3]. Inflammatory cell infiltration occurs in response to HPV tissue damage, with infiltrates consisting initially of neutrophils followed by macrophages and T lymphocytes cells. NK and NKT cells contribute to antiviral innate immune responses. NK cell activation depends on type 1 interferon and proinflammatory cytokines such as IL-12 and IL-18; these cells are able to detect decreased expression of HLA class I in infected and transformed cells [4]. Most cervical HPV infections are cleared or suppressed via cell-mediated immunity: CD4+ and CD8+ T cells are the major effector cells [4], and the Th1 response is associated with clearance of the HPV infection and regression of the cervical cancer [5]. Th2 responses are associated with cervical carcinogenesis [6].

To define patients with the highest risk of developing malignant diseases, the interaction between the host immune response and HPV infection must be investigated. The goal of the present study was to investigate the association of the polymorphic immune response genes, namely, the *KIR* genes, HLA classes I and II, and SNPs of cytokines, with HPV infection in Brazilian women. 

The *KIR* locus comprises an approximately 150 kb region located on chromosome 19q13.4, which encodes a group of inhibitory and activating KIR molecules. KIRs are key receptors of human natural killer (NK) cells, a subset of lymphocytes that trigger early innate immune response against infection and tumors [7].

The major histocompatibility complex (MHC), also known as the human leukocyte antigen (HLA) complex, located on chromosome 6p21.3, is the most polymorphic genetic system in mammalians and has been studied with regard to a wide variety of diseases of distinct etiology. The fundamental role of the different molecules within the MHC is antigen processing and presentation to the T-cell receptor (TCR), which is crucial for the cell interactions in cell-mediated immunity [8].

Polymorphisms of regulating regions of cytokine genes have been correlated with its production and can confer flexibility in the immune response to the viral infections and cancer biology. Five independent regions were investigated: chromosome 1: *IL10* region [9], chromosome 6: tumor necrosis factor (*TNF)* [10], chromosome 7: interleukin-6 *(IL6)* [11], chromosome 12:   interferon-gamma (*IFNG*) [12], and chromosome 19: transforming growth factor-beta (*TGFB1*) region [13].

## 2. Materials and Methods

### 2.1. Patients and Samples

Patients comprised 79 nonrelated, admixed Brazilian women from the state of Paraná in the southern region of Brazil, who were infected with HR-HPV and present cervical intraepithelial neoplasia (CIN) grade 3 (CIN3) and 150 women from the same region matched for ethnicity who were HPV-negative/normal cytology. The study protocol was approved by the ethics committee, and all selected patients signed the free and informed consent form.

In the Paraná state, the degree of the European ancestry is high (80.6%), with a small but significant contribution of African (12.5%) and Amerindian (7.0%) genes according to Probst et al. [14], and the studied populations were considered admixed. The risk of population stratification bias, due to differences in ethnic background between patients and controls, and variations of allele frequencies, according to ethnic background, were minimized by matching patients with control individuals of the same ethnic background, mean age, gender rates, and residence in the same geographical areas.

The patients were diagnosed with high-grade squamous intraepithelial lesion (HSIL) by cytologic smears, CIN3 by histopathology, and also with HR-HPV. 

#### 2.1.1. Cytology and Histopathology

The cervical and endocervical material was collected with the aid of an Ayre spatula and a cytobrush for cervical smears and for PCR amplification (suspended in 1 mL of 0.9% NaCl solution and stored at −20°C until analysis). The cytological smears were evaluated and reported according to the Bethesda system as atypical squamous cells of undetermined significance (ASC-US); atypical squamous cells of undetermined significance, which cannot exclude a high-grade squamous intraepithelial lesion (ASC-H); low-grade squamous intraepithelial lesion (LSIL); high-grade squamous intraepithelial lesion (HSIL); *in situ* or invasive adenocarcinoma (ISCC); or invasive squamous cell carcinoma (SCC). 

The cytological criteria for HSIL diagnosis adopted were squamous cells, either isolated or present in small fragments with fewer than ten cells. The cells were the length of the metaplastic cells, showing an increase in the proportion in the nuclear area. The nuclear irregularities, including hyperchromasia, chromatic clustering, irregularity, thickening, or multinucleation, were also used as important cytological criteria [15]. The histopathology findings of biopsy samples were classified as CIN grades I, II, or III, microinvasive or invasive squamous cell carcinoma, or *in situ* or invasive adenocarcinoma. The histological criteria for CIN were the failure of maturation of the squamous epithelium, nuclear hyperchromasia, and an increased nucleus/cytoplasm ratio. The intensity of these criteria was used to stage the degree of CIN or carcinomas [16]. The HSIL cytological cases included in the present study were confirmed as CIN III by histopatnology.

#### 2.1.2. HPV Molecular Detection

For HPV molecular detection, genomic DNA was extracted using DNAzol (Invitrogen, Carlsbad, CA, USA). HPV polymerase chain reaction (PCR) amplification for HPV was carried out using MY09 (5′-CGTCCMAARGGAWACTGATC-3′)/MY11(5′-GCMCAGGGWCATAAYAATGG-3′), and the PCR product was electrophoresed on a 1.5% agarose gel, stained with 1 *μ*g/mL ethidium bromide, and photodocumented under UV light (approximately 450 bp). Coamplification of the human beta-globin gene (approximately 268 bp) was performed as an internal control, using primers GH20 (5′-GAAGAGCCAAGGACAGGTAC-3′) and PC04 (5′-CAACTTCATCCACG TTCACC-3′) under the same conditions as the HPV PCR. Two types of controls were also included in each reaction series: “no DNA” (negative control) and “HPV-positive DNA” (positive control) [17].

Genotyping was carried out using PCR-based restriction fragment length polymorphism analysis using *Hpy*CH4V (New England Biolabs, Inc., Ipswich, MA, USA). The following HPV genotypes were determined for this genotyping method: HR (16, 18, 31, 33, 35, 39, 45, 51, 52, 56, 58, 59, 68, 66, 73, and 82), UR—undetermined risk (26 and 53), and LR—low-risk (6, 11, 30, 34, 40, 42, 43, 44, 54, 55, 61, 62, 64, 67, 69, 70, 72, 74, 81, 83, 84, and 91). The genotypes were grouped according to the International Agency for Research on Cancer (IARC) based on the carcinogenic potential and evolutionary branch [18]. 

### 2.2. Genotyping of *KIR*, HLA, and Cytokine Genes

Genomic DNA samples were extracted from 150 *μ*L of the buffy coat obtained from 5 mL of EDTA anticoagulant peripheral blood using the EZ-DNA Kit (Biological Industries, Beit Haemek, Israel). The DNA concentration was then determined using a Qubit fluorometer (Life Technologies Corporation, Eugene, Oregon, USA).

All genotyping methods were validated using previously typed and tested reference samples. Positive and negative controls were included in all genotyping method.

#### 2.2.1. *KIR* Genes Genotyping

Fourteen *KIR* genes and one pseudogene (*KIR2DL1, KIR2DL2, KIR2DL3, KIR2DL4, KIR2DL5, KIR2DS1, KIR2DS2, KIR2DS3, KIR2DS4, KIR2DS5, KIR3DL1, KIR3DL2, KIR3DL3, KIR3DS1,* and *KIR2DP1*) were studied using an in-house polymerase chain reaction using the sequence-specific primer method (PCR-SSP) according to Martin et al. [19] and adapted by Rudnick et al. [20]. Primers were synthesized by Invitrogen (Life Technologies Corporation, Grand Island, NY, USA), and the amplified products were visualized by 2% agarose gel electrophoresis.

#### 2.2.2. HLA Classes I and II Typing

HLA classes I and II allele typing was conducted using the reverse sequence-specific oligonucleotide technique (rSSO; One Lambda Inc., Canoga Park, CA, USA) with Luminex xMap technology (Luminex Corporation, Austin, USA). HLA groups 1 (C1) and 2 (C2) of HLA-C and group Bw4 of HLA-B were defined according to Carrington and Norman [21] and Petersdorf [22].

#### 2.2.3. Genotyping of SNPs in Cytokine Genes

Sequence-specific primer PCR (PCR-SSP; One Lambda Cytokine Genotyping Primer Pack, One Lambda, CA, USA) was performed to genotype the following SNPs: *TNF −308G>A * (rs1800629)*, IFNG +874T*>*A* (rs2430561)*, IL6 −174G>C* (rs1800795)*, IL10 −1082G>A* (rs1800896), *IL10 −819C>T* (rs1800871)*, IL10 −592C>A* (rs1800872)*, TGFB −509T>C* (rs1800469), and *TGFB1 +915G>C* (rs1800471) (Table 1), according to the manufacturer's instructions.

### 2.3. Statistical Analyses

Allele, genotype, and haplotype frequencies of *KIR*, HLA, and cytokines were calculated by direct counting. Fisher's exact test and the chi-square test with Yates' correction were used for statistical comparisons. *P* ≤ 0.05 were considered significant, and *P*  values were adjusted by means of the Bonferroni correction to enable multiple comparisons. The odds ratio (OR) was calculated based on the cross product ratio and the exact 95% confidence intervals (CI) using the SISA statistical package (http://www.quantitativeskills.com/sisa/index.htm). Hardy-Weinberg equilibrium [23] was determined by calculating the expected genotype frequencies and comparing them to the observed values using Arlequin software version 3.1 (http://cmpg.unibe.ch/software/arlequin3/).

## 3. Results 

The distributions of allele frequency ratios for all analyzed genes and for *KIR* haplotype frequencies were in Hardy-Weinberg equilibrium. 

There were no significant differences between *KIR* genes frequencies in patients and controls (Table 2), and the frequency distribution was similar to that reported in another study of the same region [24]. 

There was no relationship in the frequencies of ligands (C1, C2, Bw4, and HLA-A3/11) and in the combination of KIR-HLA ligands with the HPV disease (Table 3). 

The number and type of inhibitory KIR-HLA pairs were evaluated (Table 4), and there was a greater frequency in the patients of three pairs (38.0%) and two pairs (36.6%), followed by one pair (14.1%) and four pairs (11.2%). In the controls, two pairs (46.6%) were the most frequent, followed by three (32.2%), one (11.0%), and four (10.2%) pairs. The pairs KIR3DL1-Bw4 and KIR3DL2-HLA-A3/11 were not detected in either group. Significant difference was observed between patients and controls with respect to the three pairs KIR2DL2/3-C1, KIR3DL1-Bw4, and KIR3DL2-A3/11, which were more frequent in the patients. 

**Figure 1:**
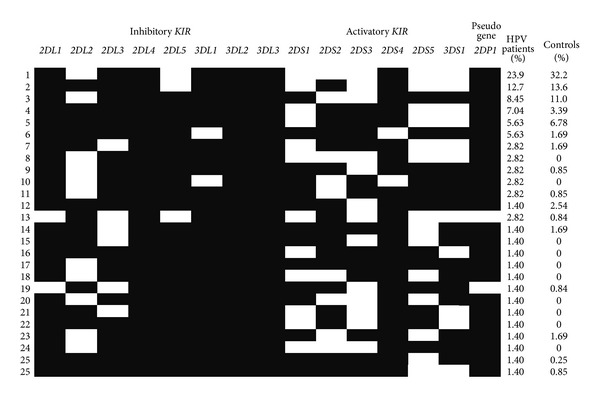
*KIR *genotypes frequencies in HPV patients and controls from Paraná, Southern Brazil.

Twenty-six KIR haplotypes were observed in HPV patients, 10 of them were not found in controls, and, otherwise, 13 others haplotypes were present only in controls (Figure 1). There were no differences between patients and controls.

No differences were observed in the distribution of HLA-A, B, and DRB1 allele groups between patients and controls (Table 5). 

Only the *HLA-A∗02-HLA-B∗51* haplotype showed a reduced frequency in HPV patients (0.006 versus 0.052, *P* = 0.0065, OR = 0.1186, and 95% CI = 0.015–0.8717) than in controls; the other six haplotypes were more frequent in the patients, but a large CI was obtained due to the small number of patients and controls (Table 6).

The cytokine allele frequencies did not differ between HPV patients and controls (Table 7), and the frequencies distribution were consistent with the results of a previous study of the same region [25]. 

Based on the genotypes, the phenotypes of cytokines production level were inferred (low, intermediate, or high). There were significant differences for the low producer phenotypes of INF-*γ*, defined by genotype AA [12], which had an increased frequency in patients (35.90 versus 29.59; *P* = 0.0221; OR = 1.81; 95% CI = 1.18–4.60), and for an intermediate producer of IL-10 (32.05% versus 48.00%; *P* = 0.0462; OR = 0.1607; 95% CI = 0.28–0.95) defined by the haplotype GCC/ACC and GCC/ATA (Table 8). The GCC/GCC genotype (high producer phenotype of IL-10) [9] was more frequent among patients (20.5%) compared with controls (12%), although this difference was not significant.

## 4. Discussion 

It is widely accepted that cofactors, including endogenous hormones and genetic factors, such as HLA and other genes related to the host immune response, may have important roles in the development of HPV-cervical lesions [26]. Inherited genetic polymorphisms within immune response genes have been shown to be associated with an increased risk of invasive cervical cancer and its immediate precursor, cervical intraepithelial neoplasia grade 3 [27]. An inappropriate innate and specific immune response may increase the risk of lesions and disease progression.

### 4.1. *KIR* and Their HLA Ligands

NK cells play an important role in innate immunity against infected and transformed cells as part of the immune surveillance process. *KIR* genes encode molecules that convey either inhibitory or activating signals (iKIR and aKIR) to NK cells and to a subset of CD8+ T cells. Binding of iKIR (designated 2DL and 3DL) to specific HLA allotypes has been clearly demonstrated and correlated to the ability to inhibit NK cytolysis of target cells bearing these HLA molecules. These interactions are remarkably complex, and synergistic relationship between these polymorphic loci may regulate NK cell-mediated immunity against viral infections [28]. 

No relationship was found between KIR genes and HPV-related cervical disease in Brazilian patients, consistent with the findings of Song et al. in Korean patients [29]. Although not significant, *KIR2DS3, KIR2DS5,* and* KIR2DL5 *were more frequent in the patient group. *KIR2DL5*, an inhibitory receptor, possesses a combination of genetic, structural, and functional features that make it unique among the KIR [30] and potentially contribute to HPV pathogenesis. *KIR3DS1* can induce a persistent, weak inflammatory reaction to HPV that results in continuous tissue injury, similar to HBV-susceptible genes [31]. Carrington et al. [32] found that the presence of the activating *KIR3DS1* is related to an increased risk of neoplasia, particularly in the absence of protective inhibitory KIR-HLA. In contrast, Arnheim et al. [33] indicated that the inhibitory allele *KIR3DL1* is associated with increased risk of CIN.

The frequencies of the ligand groups (C1, C2 group, Bw4, and HLA-A3/11) did not differ between patients and controls (data not shown). However, Madeleine et al. [34] demonstrated an association between HLA-C subtypes and squamous cell cervical cancer, and Martin et al. [27] showed that C1 (asparagine at position 80) is over represented in women with cervical cancer. In Korean women, HLA-C is associated with HPV-cervical disease: HLA-C∗03:03 confers susceptibility whereas HLA-C∗01 has a protective effect [29].

In the present work, KIR2DL1-C2 was more frequent in patients (70%) than in controls (38.5%). The strength of NK inhibition varies according to the receptor and the ligand: KIR2DL1-C2 provides a stronger inhibition than other iKIR-HLA [32, 35]. The reduced resistance to viral infections among KIR2DL1-C2-positive individuals may result from the increased inhibition of NK cells. There were significant differences for the three pairs KIR2DL2/3-C1, KIR3DL1-Bw4, and KIR3DL2-A3/11, which displayed an increased frequency in patients. This combination of iKIR and ligands could be associated with persistent inflammatory reactions that play a role in carcinogenesis. 

### 4.2. HLA and Its Association with HPV and CIN

HLA class I and class II proteins are central to host immune responses to viral infections and other pathogens. They are the most polymorphic genes in the human genome, and variations in the peptide binding groove of these proteins influence antigenic specificity. Numerous studies have evaluated the association of HLA with HPV infection and the importance of HLA in the pathogenesis of cervical neoplasia [36]. Similar to other studies [37–39], there was no association between HLA specificities and HPV infection in admixed Brazilian women from the state of Paraná. However, the *HLA-A∗02-B∗51* haplotype was associated with resistance to disease. Susceptibility to HPV infection or cervical cancer and precancerous lesion development was associated with the HLA class II: HLA-DRB1 alleles [34, 39–51]; HLA-DQB1 alleles [34, 39, 46, 49–51]; HLA-DPB1 alleles [51]; and classes I and II haplotypes [30, 34, 40–42, 48, 52, 53]. Some alleles and haplotypes had a protective effect against the progression to infection and cancer [34, 38–40, 43, 46, 47, 51, 54, 55]. In general, *HLA-DQB1∗03* increases and *DRB1∗13* decreases the risk of cervical cancer. In other Brazilian populations, Maciag et al. [44] found that HLA class II polymorphism was involved in genetic susceptibility to HPV infection and cervical cancer: *DRB1∗15:03*, *DRB1∗04:05,* and *DQB1∗06:02* alleles.

A genome-wide association study of 731.422 SNPs was performed in cervical cancer patients and controls [56]. Three independent loci in MHC region were associated with cervical cancer: the first is adjacent to the MHC class I polypeptide-related sequence A gene (MICA) (rs2516448; OR = 1.42; 95% CI = 1.31 to 1.54;  *P* = 1.6 × 10^−18^); the second is between HLA-DRB1 and HLA-DQA1 (rs9272143; OR = 0.67; 95% CI = 0.62 to 0.72;  *P* = 9.3 × 10^−24^); and the third is at HLA-DPB2 (rs3117027; OR = 1.25; 95% CI = 1.15 to 1.35;  *P* = 4.9 × 10^−8^). Previously reported associations of *B∗07:02* and *DRB1∗15:01-DQB1∗06:02* with susceptibility to *DRB1∗13:01-DQA1∗01:03-DQB1∗06:03* with protection against cervical cancer were confirmed. 

The variable results for the association between HLA and disease could be related to the differences in the distribution of HLA in the population; the disease phases (persistence or transitory HPV infection, intraepithelial neoplasia, and cancer); and HPV types. The effects of HLA polymorphisms on cervical carcinogenesis and their biological mechanisms remain unknown. Previous findings suggest a strong link between an inefficient immune response, particularly inefficient cell-mediated and innate immunity, both of which involve classes I and II HLA alleles, and susceptibility to HPV infection. HPV infections are more prevalent and more likely to persist in immunosuppressed individuals. 

### 4.3. Cytokines and HPV

Accumulating epidemiological evidence suggests that polymorphisms in cytokine genes may be involved in the etiology of cervical carcinoma [6]. Th1 cytokines such IFN-*γ* and TNF-*α* can induce a cell-mediated immune response, whereas Th2 cytokines such as IL-6 and IL-10 induce predominantly a humoral immune response and immunomodulation of the cellular response. The Th2 cytokine profile is associated with progression to cervical cancer [57]. 

In the present study, we genotyped SNPs of *TNF, IFNG, IL6, IL10,* and *TGFB1* which are multifunctional cytokine that have been implicated in inflammation, immunity, and cellular organization and have been proposed to play important roles in infection and cancer biology.

Susceptibility to infection was observed in patients with the *IFNG +874A/A* genotype, which characterized the low producer phenotype of IFN-*γ* and was more frequent among patients compared with controls. According to Telesheva et al. [58], the outcome of HPV infection is controlled by the interferon component of the immune response: a transitory course of HPV infection is characterized by increased levels of IFN-alpha and IFN-gamma, and persistent infection is related to decreased levels of IFN-alpha. 

In the current study, the *IL10 GCC/ACC* and *GCC/ATA* genotype, which characterized the intermediate producer phenotype of IL-10, were less frequent in patients, suggesting protection against disease. The IL-10 high producer phenotypes was more frequent in patients, although this increased frequency was not significant and might be related to an immunosuppressive response and development of HPV-positive cervical cancer. Serum levels of IL-10 and its expression in tumor cells are elevated in patients with cervical cancer [56]. IL-10 produced by tumor macrophages induces a regulatory phenotype in T cells and an escape mechanism of the immune response that facilitates tumor growth [59]. The SNP *IL10 −1082G>A* was not associated with susceptibility to the development of cervical cancer or HPV infection [60]. 

TGF-*β* is well known for its antiproliferative effects; however, neoplastic cells often lose their sensitivity to TGF-*β*. Iancu et al. [61] showed that in human cervical cancer, disruption of the TGF-*β* signaling pathway might contribute to the malignant progression of cervical dysplasia. In the present study, SNPs of *TGFB1 +869*, *+915* were not associated with HPV infection. 

TNF-*α*, which is secreted mainly by activated macrophages, is an extraordinarily pleiotropic cytokine that has a central role in immune homeostasis, inflammation, and host defense and could be involved in protection against HPV infection by modulating viral replication. Dysregulated TNF expression within the tumor microenvironment appears to favor malignant cell tissue invasion, migration, and ultimately metastasis [62]. Our findings are similar to those reported by Wang et al. [63], who demonstrated that there is no significant association between the *TNF −308G>A* and HPV infection or cervical cancer. However, our findings differ from those reported for the Argentina population, among whom the high producer allele *TNFA −307A* was associated with an increased risk for the development of cervical cancer [64].

*IL6* encodes a cytokine that plays important roles in the risk for cervical carcinogenesis. In the present study, there was no significant association between *IL6* and HPV-related cervical disease. However, a previous report has shown a significant association between the IL6-rs2069837 SNP and an increased risk of cervical cancer [65].

## 5. Conclusion

The genetic variability of the host contributes to the risk of HPV-related cervical disease. *KIR* genes were not associated with HPV, although some pairs of iKIR-ligands were more frequent in patients, suggesting that NK cells play a role in detrimental chronic inflammatory conditions and in carcinogenesis. HLA was associated with HPV and participated in the immune response, although its function in carcinogenesis remains unclear. The polymorphic *INFG* and *IL10* genes were associated with the outcome of HPV infection and might be indicative of impaired or invalid immune responses in patients with advanced stage lesions. Additional studies of the immune response to HPV are needed to better define the risk of developing malignant diseases associated with HPV infection.

## Figures and Tables

**Table 1:** Cytokine gene SNPs interrogated in this study.

Cytokine gene	Gene chromosome location	SNP designation in the kit	dbSNP-ID	Location
*TNF *	6p21.3	−308 G/A	rs1800629	Promoter
*IFNG *	12q14	+874 T/A	rs2430561	Intron
*IL6 *	7p21	−174 G/C	rs1800795	Promoter
*IL10 *	1q31-q32	−1082 A/G	rs1800896	Promoter
		−819 C/T	rs1800871	Promoter
		−592 C/A	rs1800872	Promoter
*TGFB1 *	19q13.1	−509 T/C (or 869 T/C)	rs1800469	Promoter
		+915 G/C	rs1800471	Exon 1

**Table 2:** Frequencies of *KIR* genes in HPV patients and controls.

*KIR *genes	HPV patients *N* = 71		Controls *N* = 118	
	*n*	%	*n*	%
*2DL1 *	68	95.8	116	98.3
*2DL2 *	35	49.3	55	46.6
*2DL3 *	63	88.7	104	88.1
*2DL5 *	43	60.6	62	52.5
*2DP1 *	68	95.8	115	97.4
*2DS1 *	30	42.2	50	42.4
*2DS2 *	37	52.1	57	48.3
*2DS3 *	26	36.6	36	30.5
*2DS4 *	65	91.5	108	91.5
*2DS5 *	28	39.4	40	33.9
*3DL1 *	65	91.5	109	92.4
*3DS1 *	31	43.6	48	40.7
*2DL4, 3DL2, 3DL3,* and *3DP1 *	71	100	118	100

*KIR* gene frequencies were similar in both the groups (*P* ≥ 0.05).

**Table 3:** Distribution of KIR and HLA ligands in HPV patients and controls.

KIR and HLA ligands	HPV patients		Controls	
	*n*	%	*n*	%
2DL1-C2	46	67.65	78	67.24
2DL1 without C2	22	32.35	38	32.75
2DL2-C1	28	80.0	43	78.18
2DL2 without C1	7	20.0	12	21.81
2DL3-C1	54	85.71	87	83.65
2DL3 without C1	9	14.29	17	16.34
3DL1-Bw4	44	67.69	73	66.97
3DL1 without Bw4	21	32.31	36	33.02
2DS1-C2	22	73.33	30	60.00
2DS1 without C2	8	26.67	20	40.00
2DS2-C1	30	81.08	45	78.94
2DS2 without C1	7	18.92	12	21.05
3DS1-Bw4	21	67.74	30	62.50
3DS1 without Bw4	10	32.26	18	37.50

Bw4: HLA-A∗23, 24, and 32; HLA-B∗08, 13, 27, 37, 44, 51, 52, 53, 57, and 58.

Group C1: HLA-C∗01, 03, 07, 08, 12, 14, and 16.

Group C2: HLA-C∗02, 04, 05, 06, 07, 15, 17, and 18.

Difference was not observed (*P* ≥ 0.05).

**Table 4:** Combinations of inhibitory KIR-HLA pairs and their frequencies in HPV and control Brazilian women from Paraná, Southern Brazil.

Number of pairs	KIR-HLA	HPV patients *n* (%)	Control *n* (%)
1 pair	2DL2/3-C1	3 (30.0)	7 (61.5)
	2DL1-C2	7 (70.0)	4 (38.5)

2 pairs	2DL2/3-C1, 3DL1-Bw4	12 (46.2)	16 (34.5)
	2DL2/3-C1, 2DL1-C2	7 (26.9)	14 (29.0)
	2DL1-C2, 3DL1-Bw4	4 (15.4)	11 (23.6)
	2DL1-C2, 3DL2-A3/11	2 (7.7)	4 (9.1)
	2DL2/3-C1, 3DL2-A3/11	1 (3.9)	2 (3.7)

3 pairs	2DL1-C2, 2DL2/3-C1, 3DL1-Bw4	14 (51.9)	25 (76.3)
	2DL2/3-C1, 3DL1-Bw4, 3DL2-A3/11^a^	7 (25.9)	2 (5.3)
	2DL1-C2, 2DL2/3-C1, 3DL2-A3/11	6 (23.0)	5 (15.8)
	2DL1-C2, 3DL1-Bw4, 3DL2-A3/11	0 (0)	1 (2.6)

4 pairs	2DL1-C2, 2DL2/3-C1, 3DL1-Bw4, 3DL2-A3/11	8 (100)	12 (100)

^a^ *P* = 0.025; OR = 3.42; 95% CI = 2.45–18.22.

**Table 5:** HLA allele frequencies in HPV patients and control groups.

HLA-A allele types			HLA-B allele types			HLA-DRB1 allele types		
	HPV patients	Controls		HPV patients	Controls		HPV patients	Controls
	*N* = 156	*n* = 300		*n* = 156	*n* = 300		*n* = 156	*n* = 300
	*n* (*f*%)	*n* (*f*%)		*n* (*f*%)	*N* (*f*%)		*n* (*f*%)	*n* (*f*%)
**A∗01**	14 (9.0)	31 (10.3)	**B∗07**	8 (5.1)	17 (5.7)	**DRB1∗01**	20 (12.8)	51 (17.0)
**A∗02**	37 (23.7)	67 (22.3)	**B∗08**	11 (7.0)	15 (5)	**DRB1∗03**	17 (10.9)	23 (7.7)
**A∗03**	21 (13.5)	29 (9.7)	**B∗13**	3 (1.9)	6 (2)	**DRB1∗04**	8 (5.1)	25 (8.3)
**A∗11**	11 (7.0)	21 (7.0)	**B∗14**	9 (5.8)	14 (4.7)	**DRB1∗07**	21 (13.5)	33 (11.0)
**A∗23**	7 (4.5)	13 (4.3)	**B∗15**	22 (14.1)	26 (8.7)	**DRB1∗08**	10 (6.4)	17 (5.7)
**A∗24**	20 (12.8)	31 (10.3)	**B∗18**	6 (3.8)	21 (7)	**DRB1∗09**	3 (19.0)	4 (1.3)
**A∗25**	2 (1.2)	7 (2.3)	**B∗27**	6 (3.8)	10 (3.3)	**DRB1∗10**	6 (3.8)	7 (2.3)
**A∗26**	2 (1.2)	13 (4.3)	**B∗35**	18 (11.5)	39 (13)	**DRB1∗11**	27 (17.3)	34 (11.3)
**A∗29**	8 (5.1)	14 (4.7)	**B∗37**	2 (1.3)	4 (1.3)	**DRB1∗12**	4 (2.6)	6 (2.0)
**A∗30**	12 (7.7)	15 (5.0)	**B∗38**	2 (1.3)	3 (1)	**DRB1∗13**	17 (10.9)	47 (15.7)
**A∗31**	6 (3.8)	13 (4.3)	**B∗39**	5 (3.2)	10 (3.3)	**DRB1∗14**	8 (5.1)	14 (4.7)
**A∗32**	2 (1.3)	9 (3.0)	**B∗40**	5 (3.2)	15 (5)	**DRB1∗15**	8 (5.1)	25 (8.3)
**A∗33**	3 (1.9)	14 (4.7)	**B∗41**	1 (0.6)	6 (2)	**DRB1∗16**	7 (4.5)	14 (4.7)
**A∗34**	1 (0.6)	1 (0.3)	**B∗42**	1 (0.6)	10 (0.3)			
**A∗66**	1 (0.6)	3 (1.0)	**B∗44**	21 (13.5)	34 (11.3)			
**A∗68**	9 (5.8)	16 (5.3)	**B∗45**	4 (2.6)	5 (1.7)			
			**B∗48**	1 (0.6)	1 (0.3)			
			**B∗49**	4 (2.6)	9 (3.0)			
			**B∗50**	2 (1.3)	8 (2.7)			
			**B∗51**	12 (7.7)	25 (8.3)			
			**B∗52**	4 (2.6)	5 (1.7)			
			**B∗53**	1 (0.6)	10 (3.3)			
			**B∗55**	1 (0.6)	3 (1.0)			
			**B∗57**	4 (2.6)	9 (3.0)			
			**B∗58**	3 (1.9)	4 (1.3)			

*N*: number of alleles; *n*: number of individuals; *f*%: alleles frequencies.

Difference was not observed between both groups (*P* ≥ 0.05).

**Table 6:** HLA haplotype frequencies with significant differences between HPV patients and controls.

Haplotypes	Patients *n* (hf)	Controls *n* (hf)	*P*	OR	CI
*HLA-A∗02*–*HLA-B∗51 *	1 (0.006)	16 (0.052)	0.006	0.1186	0.015–0.8717
*HLA-A∗03*–*HLA-DRB1∗11 *	7 (0.043)	1 (0.003)	0.002	150.828	1.712–115.230
*HLA-B∗14*–*HLA-DRB1∗13 *	5 (0.032)	1 (0.003)	0.017	108.906	1.146–85.504
*HLA-B∗15*–*HLA-DRB1∗07 *	5 (0.029)	1 (0.003)	0.017	108.906	1.146–85.505
*HLA-B∗15*–*HLA-DRB1∗11 *	7 (0.048)	1 (0.003)	0.002	150.828	1.712–115.230
*HLA-B∗44*–*HLA-DRB1∗01 *	6 (0.036)	2 (0.005)	0.018	49.028	1.188–29.885
*HLA-B∗44*–*HLA-DRB1∗11 *	7 (0.048)	1 (0.003)	0.002	150.828	1.712–115.230

*n*: haplotype numbers; hf: haplotype frequencies (%); *P*: *P* value; OR: odds ratio; CI (95%): 95% confidence interval.

**Table 7:** Cytokines alleles and genotypes frequencies in HPV patients and controls.

Cytokine alleles and genotypes	HPV patients *N* = 79 *n* (*f*%)	Controls *N* = 101 *n* (*f*%)	Cytokine alleles and genotypes	HPV patients *N* = 79 *n* (*f*%)	Controls *N* = 100 *n* (*f*%)
*TNF −308 *			*IL10 −1082 *		
*G *	139 (88.0)	174 (86.1)	*G *	50 (32.1)	72 (36)
*A *	19 (12.0)	28 (13.9)	*A *	106 (68)	128 (64)
*G/G *	62 (78.5)	73 (72.3)	*G/G *	9 (11.5)	12 (12)
*G/A *	15 (19.0)	28 (27.7)	*G/A *	32 (41.0)	48 (48)
*A/A *	2 (2.5)	0 (0)	*A/A *	37 (47.4)	40 (40)

*INFG +874 *			*IL10 −819 *		
*T *	69 (44.2)	88 (44.9)	*C *	98 (62.8)	129 (64.5)
*A *	87 (55.8)	108 (55.1)	*T *	58 (37.2)	71 (35.5)
*T/T *	19 (24.4)	19 (19.4)	*C/C *	31 (39.8)	42 (42)
*T/A *	31 (39.7)	50 (51.0)	*C/T *	36 (46.2)	45 (45)
*A/A *	28 (35.9)	29 (29.6)	*T/T *	11 (14.1)	13 (13)

*IL6 −174 *			*IL10 −592 *		
*G *	116 (73.4)	132 (65.4)	*C *	98 (62.8)	129 (64.5)
*C *	42 (26.6)	70 (34.7)	*A *	58 (37.2)	71 (35.5)
*G/G *	44 (55.7)	45 (44.6)	*C/C *	31 (39.8)	42 (42)
*G/C *	28 (35.4)	42 (41.6)	*C/A *	36 (46.2)	45 (45)
*C/C *	7 (8.9)	14 (13.9)	*A/A *	11 (14.1)	13 (13)

*TGFB1 *+*869 *			*TGFB1 +915 *		
*T *	89 (56.3)	108 (54)	*G *	141 (89.2)	190 (95)
*C *	69 (43.7)	92 (46)	*C *	17 (10.7)	10 (5)
*T/T *	26 (32.9)	25 (25)	*G/G *	63 (79.8)	90 (90)
*T/C *	37 (46.8)	58 (58)	*G/C *	15 (19.0)	10 (10)
*C/C *	16 (20.3)	17 (17)	*C/C *	1 (1.3)	0 (0)

*n*: number of observed alleles and genotypes; *f*%: allele and genotype frequencies.

Difference was not observed between both groups (*P* ≥ 0.05).

**Table 8:** Expected phenotype frequencies according to genotypes for the cytokines TNF-*α*, IFN-*γ*, IL-6, IL-10, and TGF-*β*1.

Phenotypes	Genotypes	Patients (*N* = 79) *n* (%)	Controls (*N* = 101) *n* (%)
	*TNF *		
Low	*G/G *	62 (78.48)	73 (72.28)
High	*G/A *	17 (21.52)	28 (27.72)
	*A/A *		

	*IL6 *		
High	*G/G *	72 (91.14)	87 (86.14)
	*G/C *		
Low	*C/C *	7 (8.86)	14 (13.86)

	*INFG *		
High	*T/T *	19 (24.36)	19 (19.39)
Intermediate	*T/A *	31 (39.74)	50 (51.02)
Low^b ^	*A/A *	28 (35.90)	29 (29.59)

	*IL10 *		
High	*GCC/GCC *	16 (20.51)	12 (12.00)
Intermediate^a^	*GCC/ACC *	25 (32.05)	48 (48.00)
	*GCC/ATA *		
Low	*ACC/ACC *	37 (47.44)	40 (40.00)
	*ACC/ATA *		
	*ATA/ATA *		

	*TGFB1 *		
High	*T/T G/G *	52 (65.82)	78 (78.00)
	*T/C G/G *		
Intermediate	*T/C G/C *	22 (27.85)	17 (17.00)
	*C/C G/G *		
	*T/T G/C *		
Low	*C/C G/C *	5 (6.33)	5 (5.00)
	*C/C C/C *		
	*T/T C/C *		
	*T/C C/C *		

*n*: number of excepted phenotypes according to genotypes.

%: frequencies.

^a^ *P* = 0.046; OR = 0.1607; 95% CI = 0.276–0.947.

^b^ *P* = 0.022; OR = 1.81; 95% CI = 1.178–4.604.
